# AnkFall—Falls, Falling Risks and Daily-Life Activities Dataset with an Ankle-Placed Accelerometer and Training Using Recurrent Neural Networks

**DOI:** 10.3390/s21051889

**Published:** 2021-03-08

**Authors:** Francisco Luna-Perejón, Luis Muñoz-Saavedra, Javier Civit-Masot, Anton Civit, Manuel Domínguez-Morales

**Affiliations:** 1 Architecture and Computer Technology Department, ETSII-EPS, University of Seville, 41004 Sevilla, Spain; lmsaavedra@us.es (L.M.-S.); jcivit@atc.us.es (J.C.-M.); civit@us.es (A.C.); mjdominguez@us.es (M.D.-M.); 2 Robotics and Technology of Computers Laboratory, University of Seville, 41004 Sevilla, Spain; 3 Research Institute of Computer Engineering (I3US), University of Seville, 41004 Sevilla, Spain

**Keywords:** accelerometer, deep learning, embedded system, fall detection, wearable, recurrent neural networks

## Abstract

Falls are one of the leading causes of permanent injury and/or disability among the elderly. When these people live alone, it is convenient that a caregiver or family member visits them periodically. However, these visits do not prevent falls when the elderly person is alone. Furthermore, in exceptional circumstances, such as a pandemic, we must avoid unnecessary mobility. This is why remote monitoring systems are currently on the rise, and several commercial solutions can be found. However, current solutions use devices attached to the waist or wrist, causing discomfort in the people who wear them. The users also tend to forget to wear the devices carried in these positions. Therefore, in order to prevent these problems, the main objective of this work is designing and recollecting a new dataset about falls, falling risks and activities of daily living using an ankle-placed device obtaining a good balance between the different activity types. This dataset will be a useful tool for researchers who want to integrate the fall detector in the footwear. Thus, in this work we design the fall-detection device, study the suitable activities to be collected, collect the dataset from 21 users performing the studied activities and evaluate the quality of the collected dataset. As an additional and secondary study, we implement a simple Deep Learning classifier based on this data to prove the system’s feasibility.

## 1. Introduction

Among most events related to the gait study, fall detection clearly stands out. These events can lead to severe injuries and sometimes chronic problems or even death. Its importance grows as users age, becoming one of the most important causes of morbidity and mortality worldwide among the elderly according to the World Health Organization (WHO) [1]. The obtained results estimate that around 28% to 35% of people over 65 fall at least one time per year. Additionally, this rate increments in people who suffered falls in the past. Key factors regarding falls include not only physical variables but also psychological aspects, such as fear of falling again. These aspects condition the gait of these persons, leading to higher falling risks. All these factors are correlated with the person’s way of walking.

After a fall event, it is very important to have a quick response to avoid increasing the consequences of the fall. However, in many occasions, elderly people live alone or spend too much time alone. In addition, in exceptional situations, such as the current global pandemic caused by COVID-19, we must significantly reduce mobility and contact with this population sector, since it is the most sensitive to these diseases. This is one of the reasons why automatic fall detection systems have become very important in recent years and especially in the current pandemic situation.

There are multiple fall detection system types that achieve good results in these situations—solutions integrated into the user’s home (within the “smart home” domain) using visual sensors, telecare solutions with user interaction, mobile applications or wearable systems, among others.

As has already been explained, the responses of these systems occur only after the user falls, acting as a rapid intervention mechanism when these events occur. However, in order to continuously monitor and prevent these falls, it is very interesting to detect possible risk of fall situations, although these events are much more complex to detect. Thus, it is very important to analyze the efficiency of the system based on these three states—fall, falling risk and activity of daily-living (ADL).

In order to design a new fall-detection system, it is important to collect a large amount of data to be able to use it during the system testing phase. For this purpose, researchers usually use public datasets to save time and resources in the design and implementation of the data gathering device as this eliminates or greatly reduces the workload for the collection, filtering and labeling of the required data. Also, the more data we have, the more reliable the system results will be.

As an important point, it should be noted that there are several sensors and/or systems that can be used to detect falls. However, the most widely used sensor that is integrated in almost all fall detection systems is the accelerometer. Some works add other complementary sensors, like gyroscopes (Gyr) or magnetometers (Mag) to filter the information, but the accelerometer (Accel) is the most widely used sensor (sometimes integrated in a wearable independent device and others as part of a smartphone).

In previous works, information about fall events was collected using several users, and the processed and labelled data were made publicly available for researchers as datasets. Most of these datasets are linked with published papers that describe the collecting process. Several of these datasets are summarized in Table 1. As can be observed, there are two main tendencies—using a smartphone or a wearable device. However, almost all published works use an accelerometer.

Another important point shown in Table 1 is the sensor (or device) location. Of these works, 31% place the device in the pocket (usually those that use a smartphone), another 31% place it on the waist, 6% on the chest and 6% on the wrist. Apart from this, two works place several devices in different locations and only one places it on the lower limb. With this summary, it can be observed that 87.5% of the analyzed datasets locate the device in the upper half of the body. Only one work locates the sensor at the bottom, and another uses multiple sensors (locating one of them on the ankle).

The long-term objective of this work is to implement an integrated system to analyze events associated with gait, merging the fall detection system with other gait anomaly detection systems. To achieve this goal, the fall detection system must be near the foot, in order to be able to integrate this new system with the one developed in previous works [2,3], where the pressure sensors that measured the gait trend were located in the footwear insole. For this reason, in order to study the viability of this type of systems, we need a dataset that collects the information about falls and activities of daily living (ADL) from the foot or from the ankle.

Moreover, as detailed previously, it is very important to detect not only falls, but also falling risks. As observed in Table 1, only two of the works include falling risk activities due to the difficulties regarding data collection. But, for our case, it would be very important to include these events. So, attending to the aforementioned problems and the detailed future integration work, we need a dataset obtained from the ankle or from the foot using an accelerometer and including three types of events: Falls, Falling risks and Activities of daily living (ADL).

This dataset creation would set a precedence in the field of fall detectors and could be of great help to other researchers who work in this field. This dataset could be combined in future works with classic Machine Learning (ML) or Deep Learning (DL) techniques to automatically classify the events related to the collected data. These techniques come from the field of Artificial Intelligence (AI), and they have had (and continue to have) great success when automatically extracting the meta-characteristics of large amounts of data in order to perform these classifications correctly. Not only in this area, but also in many others, both ML and DL are used assiduously to solve this type of problem; and as a result of this fact, several studies have emerged with remarkable results [4,5,6,7,8].

**Table 1:** Datasets summary.

Dataset	Year	Participants	Sensors	Location	Classes	#Activities
Frank et al. [9]	2010	16	Accel	Waist	Pasive, ADL, Risk, Fall	5
Kerdegari et al. [10]	2012	50	Accel	Waist	ADL, Fall	20
Anguita et al. [11]	2013	30	Smartphone (Accel)	Waist	Pasive, ADL	8
Medrano et al. [12]	2014	20	Smartphone (Accel)	Pocket	ADL, Fall	8
Ojetola et al. [13]	2015	42	Accel, Gyr	Chest	ADL, Risk, Fall	15
Fall-MobileGuard [14]	2015	20	Shimmer2R (Accel)	Pocket	ADL, Fall	29
Vilarinho et al. [15]	2015	3	Smartwatch (Accel)	Hand	ADL, Fall	19
Wertner et al. [16]	2015	5	Smartwatch (Accel, Gyr)	Pocket	ADL, Fall	14
MobiAct [17]	2016	57	Smartwatch (Accel, Gyr)	Pocket	ADL, Fall	13
UniMiB SHAR [18]	2017	30	Smartwatch (Accel)	Pocket	ADL, Fall	17
UMAFall [19]	2017	17	Smartwatch, Tags (Accel, Gyr, Mag)	Ankle, Waist, Wrist, Chest	ADL, Fall	11
SisFall [20]	2017	38	Accel, Gyr	Waist	ADL, Fall	33
Rescio et al. [21]	2018	15	EMG	Lower limb muscles	ADL, Fall	6
Quadros et al. [22]	2018	22	Accel, Gyr and Mag	Wrist	ADL, Fall	12
FallDroid [23]	2018	20	Smartphone (Accel)	Waist	ADL, Fall	19
UP-Fall [24]	2019	17	Wearables (Accel), EEG, Cameras, Context-aware	Wrist, Neck, Pocket, Waist, Ankle	ADL, Fall	11

Clearly, the application of these technologies in the field of e-Health, both in the analysis of physiological signals and as diagnostic aid systems with medical images, has an enormous impact and helps to significantly reduce the workload of healthcare professionals. Several works related to this area can be found [25,26,27,28], and currently we can even find some interesting applications related to the detection of COVID-19 [29,30]. Due to the rise of these systems, it is interesting to carry out a preliminary dataset study using DL techniques in order to evaluate its quality.

Thus, summarizing the aforementioned, in this work one main fundamental objective is established: designing and developing a dataset for the detection of fall events, falling risk events and activities of daily living (ADL) using a wearable device as data collector (based on low-power microcontroller, accelerometer and Bluetooth BLE transmission), located on the user’s ankle.

As a complementary objective, but not one of the main objectives, we include a study regarding the other published datasets (16 in total) in order to extract their main characteristics and their most common activities. This information will be used to determine the activities used for our own dataset. It is important to mention that, although this is not one of the main objectives, this initial study will help to determine the most suitable activities to include in the dataset; thus, this study will help to accomplish the main objective.

Finally, as an additional objective, a dataset classification study is carried out using DL techniques based on Recurrent Neural Networks (RNN). This final objective will determine the dataset quality and, for this reason, a deep study about the different parameters used for classification purposes is carried out. However, it is important to clarify that, as this is not one of our main objectives, we are not looking for the best classification result, but just for a good classifier that justifies the correct samples’ labelling. The results that will be shown regarding this classification can be (and will be) optimized using more complex RNNs (like in [31]) in future works after certifying the correctness of the dataset.

About the device location, the ankle was chosen instead of the foot for the ease and comfort when integrating the designed prototype (larger than the final device). However, the final and future device will be miniaturized and located in the footwear in order to be combined with the gait pathology detection system implemented and presented in [3].

The rest of the paper is divided as follows—first, in the ’Materials and Methods’ Section, the characteristics of the collected dataset and the device used for this purpose are presented, as well as the recurrent neural network implemented to test the dataset quality. Next, the results obtained after training and testing the dataset collected are detailed and explained in the Results and Discussion Section. Finally, conclusions are presented.

## 2. Materials and Methods

In this section, we will present in depth the collected dataset—we will discuss the users who have participated in the data collection as well as detail the activities selected to be carried out by all of them based mainly on the analysis of previous public datasets. In addition, we will emphasize the device designed for this task—we will detail the hardware design, its firmware and the user application that receives and stores the information.

Last, but not least, we will detail the selected Recurrent Neural Network architectures used to test the quality of the dataset, as well as all the parameter configurations that have been tested.

### 2.1. Ankfall Dataset

The dataset developed for this work has been called “AnkFALL” due to the location of the data recorder device (ankle), and it is publicly available to any researcher (https://github.com/mjdominguez/AnkFall) taking into account the limitations indicated in the information web page (accessed on 8 March 2021).

This dataset is stored with version control. In this way, any modification of its content would provoke the creation of a new version; so that, when researchers download the dataset, they will be able to know which version they are using and thus be able to compare themselves with other works. At the time of publication of this work, the version of the dataset is “v1”, and it will be increased numerically as future changes are made.

This section will detail the users who have participated in the data collection, the activities carried out by each one of them and the device designed for data collection.

#### 2.1.1. Activity Set and Participants

In order to consider the best activities for our dataset, it is important to analyze the sixteen previous studies related to the actual datasets that contain activities related to gait. All these studies were detailed in the Introduction Section and collect activities of daily living (ADL), fall simulations and, some of them, falling risk activities (as detailed in Table 1).

We analyzed the three activity types recorded by all of them and included only those which are more common among the sixteen datasets. However, it was established that activities which are too dangerous to carry out or too difficult to simulate would be discarded.

- Activities of Daily Living (ADL): in this kind of activity, most of the datasets include the most common ones like walking, sitting down on a chair, getting up from a chair, crouching down, getting upstairs and getting down stairs. Those ADL activities that are only present in one or two datasets were not taken into account. So, based on this study, only those common activities were included in our activity set.
- Falling Risks: regarding these activities, it is important to mention that only two of the sixteen datasets include this kind of events due to the difficult of simulating them. These two datasets include activities like recovering after trying to sit down into the void, trying to get up, stepping down from a platform, trying to dodge an obstacle on the ground and sitting with an imaginary wall. These situations were discarded due to the danger involved when performing a simulation or the difficulties about conducting a realistic simulation. However we wanted to include falling risk situations and, according to the most common situations among elderly, the risk activities included in our study were: tripping over an obstacle and walking with an improper weight change due to dizziness.
- Falls: the simulation of these activities is carried out by all the previous studies in very different ways, so a deeper analysis is needed in order to select the best activities for our dataset. A summary about fall simulation activities used in the sixteen analyzed datasets can be found in Table 2. According to this review, the most frequent fall events used in the datasets are dropping down (used in eleven of the sixteen datasets: almost 69%), tripping (used in four of the sixteen datasets: 25%), sitting into the void (used in four of the sixteen datasets: 25%) and fainting (used in four of the sixteen datasets: 25%). Thus, these four fall events are included in our dataset.

After evaluating all the datasets, we obtain a total amount of twelve activities. Another important problem we detected after evaluating the previous datasets is that the different kind of activities are unbalanced—in some datasets more than 75% of the activities are ADL; and the great majority of them do not have risk events. These problems affect the classifier system severely, as it needs balanced data in order to extract the meta-characteristics of the dataset. Thus, ADL activities were reduced in our dataset to achieve this goal. Table 3 shows the final list of activities considered for the preparation of the dataset. In this table a short description is included, as well as a detailed preparation process to record them. As can be observed, our dataset has five ADL activities, three Falling risk activities and four Fall simulations.

All these activities were performed by 21 volunteers who were previously informed and trained about the way to carry them out. Due to the difficulty to perform some of the falling risk events, the volunteers had the possibility to avoid some of them. The participants were 16 males and 5 females aged between 21 and 60, with heights between 1.60 and 1.95 m, weights between 70 and 110 kg and none of them presented any gait limitations.

#### 2.1.2. Acquisition Process

As described before, the long-term goal after this study is to develop an integrated system that records and analyzes all types gait related events. These events include not only fall events, falling risk events and ADL events; but also problems related to user balance, way of walking, plantar problems like pronation or supination, and so forth. Regarding these problems, some works have been previously developed [2,3], and the device implemented for those other works was integrated in the footwear. Thus, in order to fuse both systems (fall detector and gait analyzer) in the near future, the acquisition device was located in the ankle (as the prototype is currently too big to fit in the footwear).

Moreover, we have consulted two sets of people—the first set was composed by people who are using a similar device; and the second set includes some of the participants of the collected dataset. However, because of the difficulties of making an extensive consultation due to the current pandemic, the first set was formed by relatives and acquaintances of the authors of this manuscript.

Finally, the first set includes five persons (four of them older than 65); and the device location used by them varied according to each user—two of them wore it on the waist, one on the wrist and two on the chest. The two main problems that they commented on were discomfort when wearing it (3/5; those who wore the device on their waist did not notice discomfort), and forgetfulness (4/5; only one indicated that he did not forget it because he wears a bracelet all day). For the second set, four of the participants in the data collection were able to provide a comparative view with other devices they had used. They all agreed that the new device was more comfortable to wear. Moreover, some of the participants, at the end of the collecting process, did not remember which ankle they had the device on (as it was put over the sock).

So, according to these consultations and taking into account the proposed future works detailed in the Introduction Section, there are two main reasons why we focus the study on acquiring data on the footwear:

- This area of the body is directly influenced by risk events and falls; however, the vast majority of published datasets do not record from this position.
- This location opens the opportunity to combine the information with other sensors, such as pressure sensors, to analyze pathologies or other gait-related problems.

Regarding the forgetfulness problem, we need to analyze the focus population of the device:

- Users with many pathologies are used to wear insoles. Forgetting to use the device would be unlikely, especially if the sensors were integrated into the orthotics devices.
- The elderly usually wear a small number of shoes and, thus, it would be less common to forget the device.
- Athletes usually wear sports shoes, and therefore sensors could be installed on them.

Thus, according to this extensive study, locating the device in the footwear in a near future could be an interesting solution to avoid the inconveniences indicated before. For now, our device will be located in the ankle; but the information recorded by the accelerometer will be very similar to what will be obtained by a device located in the footwear. For this purpose, the full recording system is made up of a sensing and transmission device (placed on the ankle) and an application for monitoring and saving the received information.

**Figure 1:**
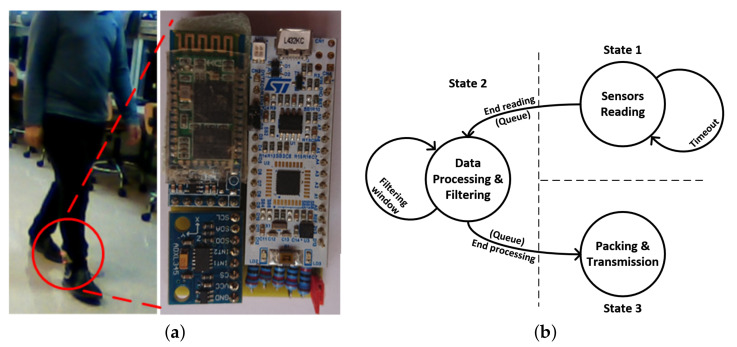
Wearable acquisition device: (**a**) wearable device placed in the ankle; (**b**) firmware’s state machine.

The wearable device is managed by a 32-bit low-power microcontroller. The full list of used components is described next (see Figure 1a):

- STMicroelectronics microcontroller (STM32L432KCU6): 32-bits ARM Cortex-M4 with floating-point unit (FPU), 80 Mhz CLK, 256 KB FLASH, 64 KB SRAM, 12-bit resolution ADC (analog-to-digital converter) up to 10 channels and up to 22 GPIOs (general purpose pins).
- ADXL345: triaxial and analog accelerometer with a resolution up to ±16 g for each axis.
- HM-10: low-energy bluetooth modem controlled by a serial port. This modem is used to transmit the collected data to the monitoring application.
- Power supply: as the main objective is the dataset collection, we used a comercial powerbank to feed the device. In a near future, with the final implementation after testing the dataset and integrating the RNN inside the embedded system, a 150–200 mAh lipo battery will be used; however, at this time, no power-consumption studies have been performed.

Regarding the device’s firmware, a real-time operating system (FreeRTOS) is used to correctly process the information (see Figure 1b) and transmit it without losing data, configuring the accelerometer with a ±16 g accuracy and positioning it in the following way—x axis was aligned with the horizontal line, y axis with the vertical, and the z axis was aligned with the march direction. The information collected from each accelerometer axis is filtered using the mean with a 10-sample window, and the resulting values are encapsulated into a frame (which contains a starting sequence, the three accelerometer filtered axis values, a checksum value to check the correct reception of the information in the computer, and an ending sequence), and transmitted at a 50 Hz rate to the computer (see Figure 1b).

On the other hand, the monitoring system plots the information received from the wearable device by the computer in real time (at 50 Hz as commented before), allowing us to visualize whether the information has been correctly captured. In the meanwhile, this data is stored locally in a csv file.

After the collecting step, the information stored for all the activities are accelerometer values (3 values, one for each axis). At this stage the different classes are not indicated yet, and we need to precisely discriminate the correct label to tag each sample individually.

**Figure 2:**
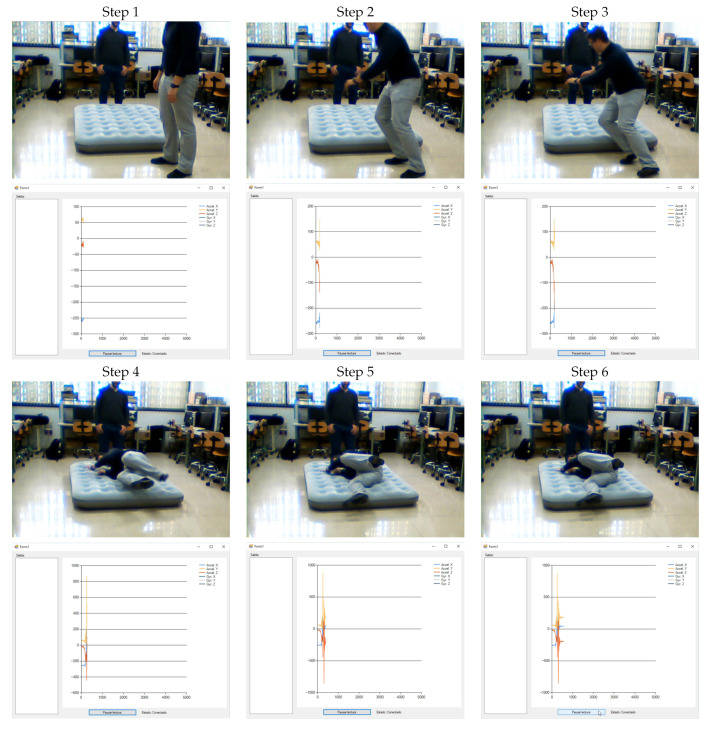
Monitoring application and videoclip during a Fall event recording sequence (in order, from step 1 to 6).

In order to perform the labelling task, a webcam records the activities while the monitoring system is plotting the information. A local script launches the monitoring application and the webcam recording tasks at the same time. Thus, the csv file (that contains the accelerometer information) and the clip recorded by the webcam share the timestamps, easing the subsequent labelling task. Therefore, we store the accelerometer information and the videoclip for every task for all users. However, for confidentiality reasons these videos are not publicly available. A recording sequence from both the monitoring application and the videoclip is shown in Figure 2.

Viewing the video in slow motion, we labelled the entire dataset sample by sample between the available classes, that is, fall, falling risk and ADL. However, those periods in which none of the three previous possibilities occurred (a section where the user does not move), were labelled with an auxiliary category called background (BKG) that corresponds to the class of other datasets named “Passive” (see Table 1). So, finally, our dataset distinguishes between four classes—BKG, ADL, FALL and RISK. The reasons why we use these classes are detailed below:

- Most studies group events to simplify the final problem. In fact, there are many studies that only classify between fall events and ADL, without addressing risk events.
- Knowing the specific user activity is not as interesting for healthcare systems as detecting dangerous anomalies like falls or falling risks.
- Using more classes requires more complex models and the systems would loose the ability to alert in real time.

### 2.2. Recurrent Neural Network Classifier

As detailed previously, the main goal of this work is the elaboration of one dataset that discretizes between falls, falling risks and activities of daily living (ADL), and the way in which the activities are selected as well as the methodology to used to perform them was described before. However, in order to evaluate the quality of the collected dataset, a DL based classification will be carried out using recurrent neural networks (RNN) in the same way as it was done in previous works with other datasets [31]. It is important to emphasize that our objective is not to optimize the final classification accuracy of the system; however, we study the different parameter combinations for the network in order to evaluate if it is possible to classify the different activities and, if this is the case, the adequacy of the collected dataset will be demonstrated. For this purpose, we need to evaluate the specificity metric—this metric determines measures of the proportion of values not belonging to a class that are correctly identified as such. So, a good result in this metric means that the different classes can be differentiated easily and, thus, the dataset collecting and labeling processes can be verified using this metric.

**Figure 3:**
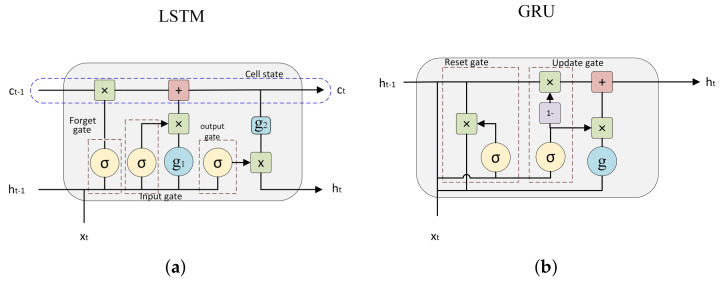
Long Short-Term Memory (LSTM) (**a**) and Gated Recurrent Units (GRU) (**b**) units.

In order to perform this task, we used Gated Recurrent Neural Network algorithms. These algorithms can automatically extract the appropriate characteristics from a temporal sequence (like our dataset) for performing the final classification. Long Short-Term Memory (LSTM) and Gated Recurrent Units (GRU) are the most used gated recurrent layers (see Figure 3), which have been demonstrated to obtain an acceptable performance in signal classification problems [32,33].

If we generalize the problem, the gait-related events require a study of acquired signals sequential/temporal characteristics, as detailed before. To contemplate this temporality there are two main approaches; by using frequency analysis (using FFT or DWT ), extracting features and combining them with a classic algorithm such as an MLP network; or by using deep learning algorithms such as RNNs. As the final future objective of his work is to create real-time systems, the feature extraction process needed in the frequency analysis method requires more computational time than working directly with RNNs (with not too many layers); therefore, for a real-time system, it is more consistent to work with RNNs.

In previous studies, we used these recurrent layers with accelerometer signals taken at the waist (using the information of other datasets), obtaining high effectiveness results and real-time classifications using low-power microcontrollers [3]. These layers implement a memory cell that retains relevant information from the analyzed sequence section, to use it in the analysis of the full sequence and, finally, to provide appropriate information for the classification problem. In summary, their operation is detailed below:

- LSTM cell: this cell consists of an input gate, a forget gate and an output gate. The first gate adds new information to the cell from the sequence sample in the current instant of time *t* and the outputs of the same layer processing for the previous instant t−1. With the same inputs, the forget gate determines the non-necessary data for the analysis of rest of the sequence. Finally, the output gate passes information from the memory that considers relevant as input to the layer processing the next instant t+1.
- GRU cell: it has only two gates (no forget gate is used), so it can add (update gate) and remove (reset gate) information from the cell, allowing all stored information to be used by the neural network throughout the sequence analysis.

**Figure 4:**
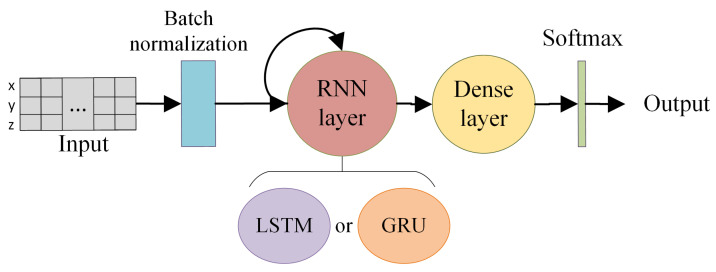
Diagram of the Gated RNN architectures assessed.

In this study two main architectures were used (see Figure 4). Both of them contain a single recurrent layer and a dense layer with four nodes. A softmax activation function is used to normalize the results in a probability distribution; and, thanks to this, both the loss calculation and the 4-class classification results can be performed. Additionally, we use a batch normalization layer during the training step to normalize the input data, allowing a faster convergence of the system. The two architectures differ in the type of RNN layer used—the first one is based on LSTM cells while the other one uses GRU cells.

#### Data Segmentation and Labelling Criteria

**Figure 5:**
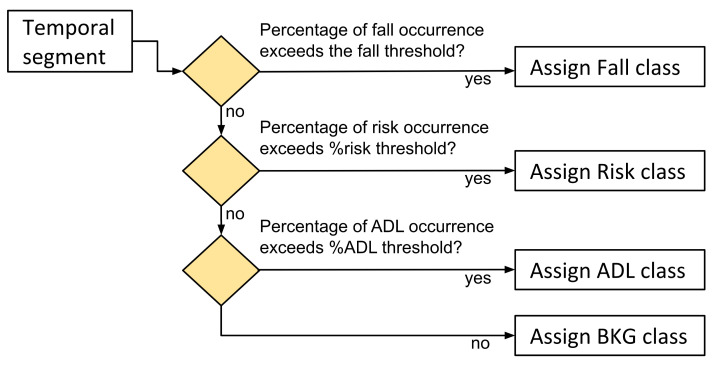
Labelling criteria based on occurrence percentage in each block.

In order to adapt each registered activity to the RNN models, we established a fixed temporal window of 64 samples, which, considering an acquisition frequency of 50 Hz, is equivalent to 1.28 s. This window is appropriate to contemplate a fall event or even risk situations [34]. Each activity was split into blocks with the same window length of 64 and was labelled with a unique category. The criteria used for labelling each block consisted of assigning it to the most relevant class whose occurrence percentage exceeds an established threshold (see Figure 5). If none of the thresholds is reached, the segment is classified as background (BKG).

In the first step of this study, we consider different threshold values for each relevant category and analyze the training results to obtain the best threshold values. In a second step, grid search is used for model optimization. The parameters considered were the number of nodes and dropout value for the recurrent layer, as well as learning rate and batch size used during training. All these tests and the obtained results will be detailed in the next section.

## 3. Results and Discussion

After analyzing all the datasets developed in recent years related to fall events and selecting the activities that must be collected for the new dataset, its final distribution is shown below.

In addition, as already indicated above, we consider that it is necessary to test our dataset using a DL classification system. In this way, we will assess whether the collected dataset is useful to future fall detection systems studies.

### 3.1. Collected Dataset

While recording the dataset, three repetitions of each activity were performed. However, due to the COVID-19 pandemic lockout, some activities could not be registered. It mainly affected activities 5 (going up and down stairs) and 8 (walking while dizzy) because they needed special considerations during the preparation. Thus, the data recollection for these activities was delayed until the other activities were recorded. While activity 8 (walking while dizzy) was performed by 10 users, activity 5 (going up and down stairs) could not be performed by any user.

So, finally, eleven activities were recorded. Moreover, as activity 5 is considered an ADL (activity of daily living), the final dataset is more balanced than our initial expectations as it contains 4 ADL activities, 4 fall activities and 3 falling risk activities.

**Figure 6:**
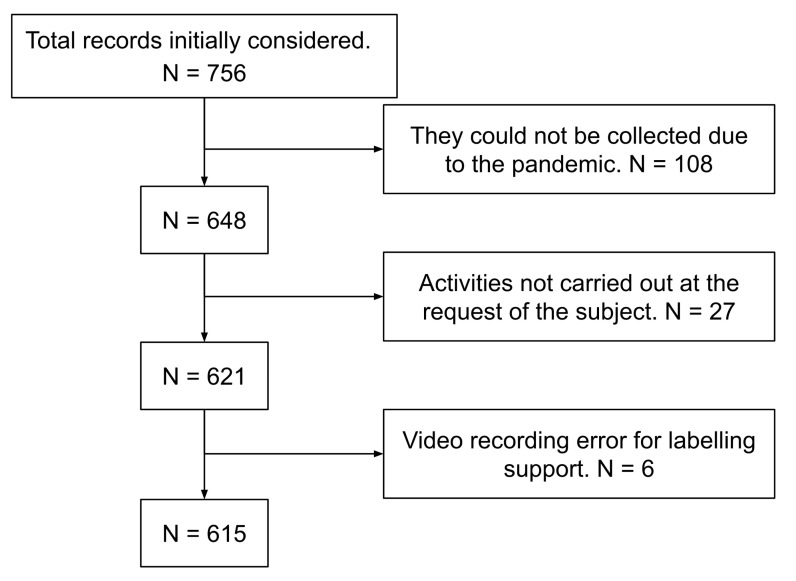
Resulting dataset.

Additionally, some participants did not carry out some activities for security reasons or at their own request and, in some cases, some errors were detected after some recordings (in particular with two activities for participant seventeen); all these reasons reduced the final amount of records. Figure 6 illustrates the process to obtain the final dataset. Finally, the total set contains 615 records.

So, after recording the AnkFALL dataset, it is important to compare it with the datasets presented in the Introduction Section. However, as there are too many differences among them, only datasets that use a wearable device with an accelerometer (not a smartphone or smartwatch) and that include fall events are taken into account. The comparison is presented on Table 4.

As can be observed in Table 4, AnkFALL registers a total number of participants and a number of activities slightly lower than average (around 30 and 19, respectively). As detailed before, the dataset is continuously growing and, in the near future, the total number of participants will increase; however, due to the pandemic lockout, no participant record has been registered since March 2020. Regarding the number of activities, AnkFALL seems to have very few, but the dataset it was designed to be as balanced as possible—as it is shown in the last column of Table 4, we can observe some goodness about AnkFALL:

- AnkFALL is the most balanced dataset among the existing ones according to the number of activities: around 36%, 28% and 36% of ADL, Risks and Falls, respectively. If we do not take into consideration AnkFall, the more balanced dataset is the one presented by Ojetola et al. [13] with 53%, 7% and 40% of ADL, Risks and Falls, respectively.
- Only two datasets from Table 1 register more Fall events than AnkFALL—the one from Ojetola et al. [13] with six different Fall activities and FallDroid [23], which has no Risk activities and uses a smartwatch.
- No other dataset has more Risk activities than AnkFALL—the datasets that have Risk activities only register one or two types.

**Figure 7:**
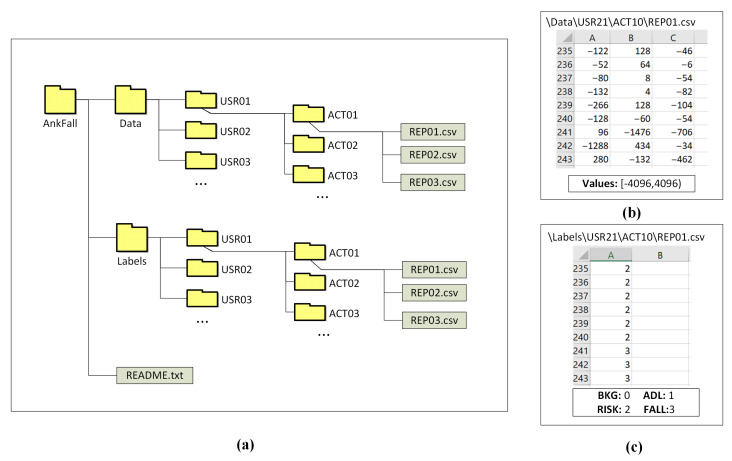
AnkFall internal structure: (**a**) Folder structure; (**b**) Data collected from user 21 during activity 10, repetition 1; (**c**) Labels for each sample of data collected during activity “(**b**)”.

Thus, the two most important things that make AnkFALL useful for researchers is that it has several Risk activities records, and it is clearly the most balanced available dataset. These two characteristics are very useful when implementing ML classification systems due to the importance of balancing the training data for obtaining good classification results without overtraining one of the classes. The internal data structure of AnkFall dataset can be observed in Figure 7.

### 3.2. Testing the Dataset

Although the main goal of this work is already achieved, it is very interesting to perform a first classification study in order to check that the information recorded and the applied pre-processing techniques are correct.

For this purpose, the Hold-Out technique has been applied, dividing the dataset in two subsets—one subset was used for training and the remaining was used for evaluation. The distribution was carried out in such a way that each subset contained data from different users, to avoid bias. Data from 5 users were randomly selected, avoiding choosing the users who had carried out the least number of activities. In the dataset segmentation process, we used a 64-sample temp window, as mentioned above, and a 25% displacement of the sample size, which corresponds to 16 temporal samples and applied this approach as a data augmentation technique: this means that there is an overlapping of 75% of the information between two consecutive temporal windows. Data distribution is shown in Table 5.

We compared the effectiveness of the classification system using different and well-known metrics: sensitivity (also known as recall), specificity, precision and F1-score [35]. This last metric is the harmonic mean of precision and sensitivity. Those metrics are presented in the next equations:

(1) $$\mathrm{Specificity}=\sum_{c}\frac{TN_{c}}{TN_{c}+FP_{c}},c\in classes$$

(2) $$\mathrm{Precision}=\sum_{c}\frac{TP_{c}}{TP_{c}+FP_{c}},c\in classes$$

(3) $$\mathrm{Sensitivity}=\sum_{c}\frac{TP_{c}}{TP_{c}+FN_{c}},c\in classes$$

(4) $$F1\text{-}\mathrm{score}=2*\frac{\mathrm{precision}*\mathrm{sensitivity}}{\mathrm{precision}+\mathrm{sensitivity}}.$$

About those metrics:

- Specificity: proportion of “true negative” values in all cases that don’t belong to this class (see Equation (1)).
- Precision: proportion of “true positive” values in all cases that have been classified as it (see Equation (2)).
- Sensitivity (or Recall): proportion of “true positive” values in all the cases that belong to this class (see Equation (3)).
- F1: It considers both the precision and the sensitivity (recall) of the test to compute the score. It is the harmonic mean of both parameters (see Equation (4)).

In the first stage of the study, the best threshold values for block labelling were analysed. We considered the values shown in Table 6.

For training our classification system, we used the best architecture considered in a previous work [31]—this architecture consists, as detailed in the previous section, of a recurrent layer followed by a dense layer and a softmax activation function. The resulting best values for thresholds were 30% for Fall events, 20% for Falling risk events and 30% for ADL, with a mean F1-score up to 0.75 and a standard deviation 0.08 after three training repetitions. The dataset distribution using the considered thresholds can be consulted in Table 7. It can be seen how, despite trying to homogenize the number of samples for each type of event, due to the short duration of risk situations and fall events, an imbalance in the number of dataset samples can be observed.

The model optimization was carried out with a grid search considering the parameter values shown in Table 8. The parameters consisted of the number of nodes of the recurrent layer, dropout rate, learning rate and batch size. This range of values was selected based on the parameters considered in previous studies [31,34].

After carrying out all the training studies with all the possibilities for each parameter, the best trained models were obtained and are shown in Table 9. In order to check the quality of these models, this table shows the values of the metrics described in Equations (1)–(4). As can be observed, two possibilities are shown in this table based on the two network types considered in this work—the first one contains LSTM units, and the second one contains GRU units.

If we analyze the results shown in Table 9, we can obtain the next conclusions for each metric:

- Specificity: we can see values over 92% that denote a high rate of true negatives among the total amount of true negatives and false positives. So, in general, this system classifies correctly the values that do not belong to each class.
- Precision and Sensitivity: these values are lower than the specificity, however both have similar values around 77–78%. These results are not bad at all, but the important difference between the specificity and these two metrics indicate that the system does not behave in the same way with all classes. Maybe one of the four classes has quite worse results than the others. In fact, if we study the system deeply, we can check that the class RISK has much worse results than the others due to two main reasons: the difficulty of distinguishing it and the alternation between RISK, ADL and BKG during the Falling risk activities. This aspect will be studied later.
- F1-score: As this metric is obtained from precision and sensitivity, it is normal that its value is similar to them. The main conclusion obtained with this value is the same that was already obtained for the previous metrics.

After presenting all the metrics, we can observe that the obtained “specificity” values are high (92–93%). This fact indicates that the system classifies the values that not belong to each class correctly. This means that the different classes can be easily differentiated and, thus, the dataset collecting and labeling processes are proven to be correct. So, this is a good metric to evaluate the quality of the collected dataset. Regarding the “precision“ metric, it depends on each class independently. But, thanks to the good specificity results, we can assume that, using more complex RNNs the classification results can be improved.

**Figure 8:**
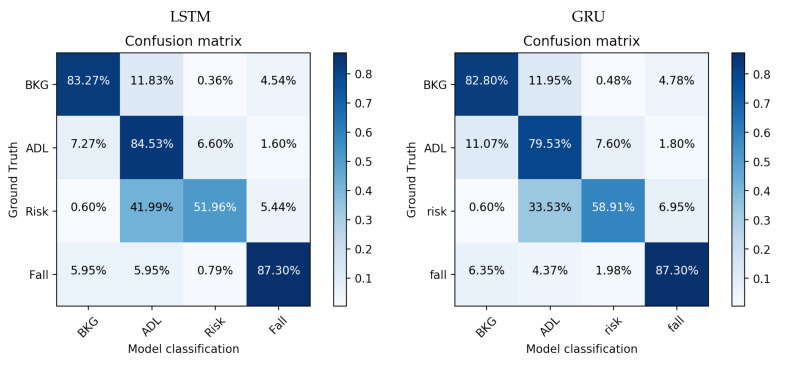
Confusion matrices for best LSTM and GRU models. Each specific box in the confusion matrix represents the percentage of samples of the class indicated by the row that have been classified as the class indicated in the column.

So, in order to corroborate the results predicted previously by evaluating the metrics, it is important to study each class independently for the two neural networks (LSTM and GRU from Table 9) selected after our initial study. For this purpose, the confusion matrices are shown in Figure 8.

This figure reveals the predicted main obstacle to be faced in the detection problem—the correct distinction of falling risk activities from activities of daily living (ADL). However, if we focus our attention on the other classes, their results are high, obtaining an 87% success rate when classifying falls. These results demonstrate that the information recorded for the AnkFALL dataset is very useful for being used in fall detection studies. The problem concerning the RISK class is not new as, in a previous work [31], a RISK class was included in the SisFALL dataset, and the conclusions were similar—the falling risk events are much more difficult to distinguish than any other event type.

**Figure 9:**
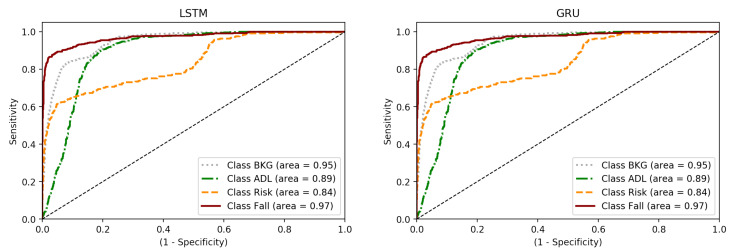
ROC curves for best LSTM and GRU hmodels.

Concerning the system ROC curves (see Figure 9), they reveal the same problem again: RISK class obtains worse results than the others. However, the area under the curve is high, revealing that, in binary classifications of the One-vs-All type, the system has the ability to distinguish each event class individually.

The curves corresponding to ADL and RISK are those with the lowest area under the curve, 90% and 80% respectively, which is correctly correlated with the results shown in the confusion matrices. Regarding the RISK class, a stagnation can be seen from a sensitivity value of 0.75, which reveals that there are samples of this class which are very complex to identify. These samples are mainly those corresponding to activity 8 (walking dizzy); so, it would be interesting to eliminate this activity from a future training study in order to corroborate these preliminary conclusions.

## 4. Conclusions

In this work, a new dataset called AnkFALL is presented. It contains labelled information from 21 participants performing 11 activities, including activities of daily-living (ADL), Falls and Falling risk activities. These activities have been carefully selected by performing a study of the different available datasets and taking into account that one of the main objectives is to obtain a dataset that contains all types of activities and that is as balanced as possible.

According to the explanations given in the introduction, the results of this work can be used in healthcare and fitness areas. Our main focus area is healthcare, and more precisely telecare services for older people who use devices to alert about emergency situations (mainly falls). Using a device placed in the footwear may prevent forgetfulness and improve wearing comfort and, thanks to this dataset, researchers can start to develop footwear-placed fall detectors. Although this is our main interest area, these principles can be easily applied to the fitness field too.

To record the dataset, a personalized wearable device, based on a low-power microcontroller, an accelerometer and a Bluetooth low energy interface, has been designed and implemented to transmit the information from the user’s ankle; on the other hand, a computer application has been implemented for the reception, visualization and storage of the received data. The labelling task has been thoroughly carried out by checking the received information sample by sample with the synchronized videoclips recorded while performing the activities.

The final result is the first dataset of its kind that collects information from the ankle, and it is also the most balanced fall-detection dataset among of all the datasets currently described in the literature. Also, this dataset includes a type of activity (falling risk) that is only available in a small subset of the available datasets.

To verify the quality of the dataset, a deep learning classifier system based on recurrent neural networks has been designed and implemented for the classification of the four classes in this dataset—BKG, ADL, Fall and Risk. A detailed study is carried out with multiple variations of thresholds, learning rates, batch sizes, number of nodes and dropout; obtaining, in combination, more than 600 different neural networks that have been trained for this purpose. The best result for each parameter is presented and the final classification results are shown. The obtained specificity values (between 92% and 93% depending on the implementation) indicate that the system classifies correctly the values that do not belong to each class. This means that the different classes can be differentiated easily, so the quality of the dataset is proven by this result. Regarding the precision, it depends on each class but, thanks to the specificity results, we can assume that, using more complex RNNs the classification results can be improved.

Moreover, the classification results show that the main difficulty is detecting falling risk events—this is mainly due to the speed at which they occur and the combination with other ADL activities; not surprisingly, most of the datasets do not include this type of activities and most of the research studies do not include them either.

For future versions this dataset needs to be expanded with a larger number of participants. Moreover, after testing the dataset, we have observed that the information collected for one of the activities has several errors and this affects the classification results; thus, in the next versions of the dataset we need to evaluate whether the instructions given for the participants were clear enough and if this activity is essential or not. Finally, in order to obtain a better adequacy of the activities collected for the dataset, we have started to apply “Explainable Deep Learning” techniques to the collected data; we hope that these techniques will help us improve the next versions of the dataset.

## Figures and Tables

**Table 2:** Fall types considered in previous datasets. Reference [11] is empty as it does not consider any fall event.

	[9]	[10]	[11]	[12]	[13]	[14]	[15]	[16]	[17]	[18]	[19]	[20]	[21]	[22]	[23]	[24]
With flexed knees		✓					✓		✓							
Sitting on empty		✓		✓			✓									✓
Base on wall					✓		✓									
Fall as subject prefers		✓													✓	
From standing (drop down)	✓			✓	✓	✓	✓		✓	✓	✓			✓	✓	✓
Pushing															✓	
Improper weight shift															✓	
Trip							✓	✓				✓			✓	
Walking with improper weight shift															✓	
Sitting with imaginary wall															✓	
Walking and slip								✓				✓			✓	
Syncope/fainting/falling asleep				✓		✓						✓			✓	
Trying to get up (seat)								✓		✓						
Trying to sit down (seat)												✓				
Lying down on a bed													✓			
Lying initially on the knees						✓			✓							✓
Rolling out of bed						✓										
With compensation strategies to prevent the impact				✓						✓						
With contact with an obstacle before hitting the ground				✓						✓						
Steing down from platform							✓									

**Table 3:** List of recorded activities. Last column indicates the mean execution time (in seconds) for each activity.

# Activity	Activity Description	Activity Steps	Time
Act. 1	The subject walks.	0. Standing; 1. Walk; 2. Stop	17
Act. 2	The subject sits in a chair.	0. Standing in front of a chair; 1. Turn around; 2. Sit	7
Act. 3	The subject gets up from a chair.	0. Sitting on a chair; 1. Get up; 2. Stand	6
Act. 4	The subject crouches down with the intention of touching the ground.	0. Standing; 1. Crouch down; 2. Return to upright position; 3. Stand	6
Act. 5	The subject goes up and down stairs.	0. Standing; 1. Go up stairs; 2. Stop; 3. Turn around; 4. Go down stairs; 5. Stand	X
Act. 6	The user trips over with the left foot.	0. Standing; 1. Walk; 2. Trip over a creased carpet with the left foot; 3. Regain balance; 4. Walk; 5. Stand	8
Act. 7	The user trips over with the right foot.	0. Standing; 1. Walk; 2. Trip over a creased carpet with the right foot; 3. Regain balance; 4. Walk; 5. Stand	8
Act. 8	The user walks while dizzy.	0. Standing; 1. Turn on itself several turns; 2. Walk; 3. Stop	19
Act. 9	The user falls backwards while sitting on the void.	0. Stand; 1. Try to sit; 2. Fall; 3. Stay still	6
Act. 10	The subject trips over and falls forward.	0. Stand; 1. Walk; 2. Trip over a creased carpet with the left foot; 3. Fall; 4. Stay still	10
Act. 11	The subject falls to the left (faint).	0. Stand; 1. Fall without resisting to the left; 2. Stay still	5
Act. 12	The subject falls to the right (faint).	0. Stand; 1. Fall without resisting to the right; 2. Stay still	5

**Table 4:** Comparison between AnkFALL and the most similar datasets (from Table 1). The last column indicates the number of activities from each type (in order: activities of daily living (ADL), Risk, Fall).

Dataset	Year	#Users	Sensors	Location	Classes	#Activities	Balance
Frank et al. [9]	2010	16	Accel	Waist	Pasive, ADL, Risk, Fall	5	3-1-1
Kerdegari et al. [10]	2012	50	Accel	Waist	ADL, Fall	20	17-0-3
Ojetola et al. [13]	2015	42	Accel, Gyr	Chest	ADL, Risk, Fall	15	8-1-6
SisFall [20]	2017	38	Accel, Gyr	Waist	ADL, Fall	33	29-0-4
Quadros et al. [22]	2018	22	Accel, Gyr and Mag	Wrist	ADL, Falls	12	11-0-1
AnkFALL	2020	21	Accel	Ankle	BKG, ADL, Risk, Fall	11	4-3-4

**Table 5:** Participant distribution for each subset using the hold-Out method.

Subsets	Users	# Activities
Train	1, 2, 3, 4, 6, 7, 11, 12, 13, 14, 15, 16, 17, 18, 19, 20	463
Validation	5, 8, 9, 10, 21	152

**Table 6:** Occurrence thresholds values analyzed for each class.

Threshold	Set of Values
ADL	0.3, 0.4, 0.5, 0.6, 0.7
RISK	0.2, 0.3, 0.4, 0.5
FALL	0.1, 0.2, 0.3, 0.4

**Table 7:** Dataset distribution for each subset with best labelling thresholds.

	Samples Per Class				
Subsets	Total	ADL	BKG	Risk	Fall
Train	9099	2064	4587	1165	1283
Validation	3172	837	1500	331	504

**Table 8:** Grid search values for exhaustive parameters optimization.

Parameters	Set of Values for Grid Search
Learning rate	0.0005, 0.001, 0.0015, 0.002
Batch size	32, 48, 64
Number of nodes	24, 32, 40
Dropout	0, 0.2, 0.35

**Table 9:** Best results obtained after grid search optimization.

RNN	RNN	Learn.	Batch	RNN				
Architecture	Nodes	Rate	Size	Drop.	Precision	Specificity	Sensitivity	F1-Score
One LSTM layer	32	0.002	32	0.2	0.780	0.928	0.768	0.774
One GRU layer	40	0.002	32	0.2	0.762	0.924	0.771	0.766

## Data Availability

Public available **AnkFall Dataset** on https://github.com/mjdominguez/AnkFall.
